# Co-Microencapsulation of Islets and MSC CellSaics, Mosaic-Like Aggregates of MSCs and Recombinant Peptide Pieces, and Therapeutic Effects of Their Subcutaneous Transplantation on Diabetes

**DOI:** 10.3390/biomedicines8090318

**Published:** 2020-08-31

**Authors:** Yusuke Mochizuki, Ryo Kogawa, Ryuta Takegami, Kentaro Nakamura, Akira Wakabayashi, Tadashi Ito, Yasuhiro Yoshioka

**Affiliations:** Bioscience and Bioengineering Laboratory, Research and Development Headquarters, FUJIFILM Corporation, Kanagawa 258-8577, Japan; ryo.kogawa@fujifilm.com (R.K.); ryuta.takegami@fujifilm.com (R.T.); kentaro.a.nakamura@fujifilm.com (K.N.); akira.wakabayashi@fujifilm.com (A.W.); tadashi.ito@fujifilm.com (T.I.); msksm.yoshioka@gmail.com (Y.Y.)

**Keywords:** CellSaic, ADSC, microencapsulation, xenotransplantation, islet, subcutaneous, recombinant peptides, type I diabetes

## Abstract

The subcutaneous transplantation of microencapsulated islets has been extensively studied as a therapeutic approach for type I diabetes. However, due to the lower vascular density and strong inflammatory response in the subcutaneous area, there have been few reports of successfully normalized blood glucose levels. To address this issue, we developed mosaic-like aggregates comprised of mesenchymal stem cells (MSCs) and recombinant peptide pieces called MSC CellSaics, which provide a continuous release of angiogenic factors and anti-inflammatory cytokines. Our previous report revealed that the diabetes of immunodeficient diabetic model mice was reversed by the subcutaneous co-transplantation of the MSC CellSaics and rat islets. In this study, we focused on the development of immune-isolating microcapsules to co-encapsulate the MSC CellSaics and rat islets, and their therapeutic efficiency via subcutaneous transplantation into immunocompetent diabetic model mice. As blood glucose level was monitored for 28 days following transplantation, the normalization rate of the new immuno-isolating microcapsules was confirmed to be significantly higher than those of the microcapsules without the MSC CellSaics, and the MSC CellSaics transplanted outside the microcapsules (*p* < 0.01). Furthermore, the number of islets required for the treatment was reduced. In the stained sections, a larger number/area of blood vessels was observed around the new immuno-isolating microcapsules, which suggests that angiogenic factors secreted by the MSC CellSaics through the microcapsules function locally for their enhanced efficacy.

## 1. Introduction

Islet transplantation is known to be a promising approach to treat type I diabetes [1]. Nevertheless, several problems remain to be addressed, such as destruction by autoimmunity and allogeneic rejection, hypotrophy and hypoxia of transplanted islets, and the toxic effects of immunosuppressive agents [2]. Additionally, the limited supply of donor pancreases and suboptimal yields of islets isolated from the pancreas also remain crucial problems [2,3].

One approach to solve these problems is the microencapsulation of islets [4,5]. With this technology, islets are encapsulated with a biocompatible material which functions as a vehicle to provide an immuno-isolated environment, replicating the native micro- and macro-environments surrounding islets [2]. This immuno-isolation template could potentially prevent allogeneic or xenobiotic rejections, which would reduce the use of immunosuppressive agents. Over and above that, this strategy might even solve the problem of the donor pancreas shortage by enabling the transplantation of heterologous islets and insulin-secreting cells [6].

Nevertheless, stable insulin withdrawal and long-term functional maintenance upon transplantation of microencapsulated islets remain challenging issues in the treatment of type I diabetes [7]. This is due to the early dysfunction of the islets caused by inflammatory responses by foreign body reactions and the lack of blood vessels around transplanted sites [8,9]. The subcutaneous transplantation of microencapsulated islets is known to be especially difficult because early dysfunction of islets can easily be caused by a lower number of blood vessels and stronger foreign body reactions [10,11]. Consequently, there have been only a few reports of the successful normalization of blood glucose levels via subcutaneous transplantation [11]. Still, subcutaneous transplantation is an attractive approach for microencapsulated islets in terms of the transplantation site because of their safety and ease of transplantation, availability of retrieval and biopsy of transplants, and large transplant volume capacities [10].

To this end, we developed a cell transplantation platform called “CellSaic”, which enhances the viability of transplanted cells. “CellSaic” is a term coined by combining the words “cell” and “mosaic”. A CellSaic is a three-dimensional structure made by combining cells with a new bioresorbable material—a recombinant protein (RCP)—for medical uses. The RCP is a recombinant protein produced by the yeast *Pichia pastoris*. It differs from conventional animal collagens as there is no risk of infections such as bovine spongiform encephalopathy [12]. In contrast to spheroids, the spaces between the petaloid RCP pieces of the CellSaic allow for the sufficient penetration of substances into the cells, which effectively prevents cell death [13]. Mesenchymal stem cells (MSCs) are promising options as the cells comprising CellSaics for subcutaneous transplantation since they have revascularization, anti-inflammatory, and immunomodulatory abilities [14,15,16,17] and are known to enhance islet function when co-transplanted with them [18,19]. Our previous study demonstrated the continuous release of angiogenic factors and anti-inflammatory cytokines via a combination of MSCs and CellSaics (MSC CellSaics) [13,20]. Furthermore, the subcutaneous co-transplantation of the MSC CellSaics and islets into immunodeficient mice was shown to have higher therapeutic efficiency compared to co-transplantation of MSCs and islets [13]. In order to expand this scheme for immunocompetent mice, the immune response against transplanted islets must be avoided.

To provide a feasible immuno-isolated environment for islets, there are three requirements, based on the literature [4,5,6,9,21]: (1) the ability to block humoral and cellular immunity; (2) allowing the permeation of small molecules such as insulin and glucose; (3) physical resistance that does not break at the transplantation site. The microencapsulation of islets has been shown to meet these requirements, and many techniques have been reported [7,11,21,22,23,24,25,26,27,28]. As a microencapsulating material for establishing immuno-isolated environments, alginate polymer is often used because it is inexpensive, biocompatible, and can create a gel easily at room temperature in aqueous solution with divalent ions. Although alginate polymer contains a large amount of impurities after ordinary production because it is a natural polymer, it is known that these impurities can be reduced by increasing the degree of purification [21]. Additionally, it is known that alginate gels’ features depend on their cross-linking agents, where divalent salts, such as Ca^2+^ [22] and Ba^2+^ [23], and polycations, such as poly-L-ornithine (PLO) [24], have been reported as suitable cross-linking agents. The physical strength and permeability of substances differ depending on the crosslinker, so it is necessary to adopt one that is suitable for the cell type.

In this report, we developed new immuno-isolating microcapsules which co-encapsulated the MSC CellSaics and rat islets (MSC CellSaic(in)) with alginate polymer and PLO as crosslinkers, and they were transplanted subcutaneously into immunocompetent diabetic model mice. The normalization rates of blood glucose levels (%NBGL) for the following 28 days were compared with those of the microcapsules without the MSC CellSaics (MSC CellSaic(none)) and the MSC CellSaics transplanted outside the microcapsules (MSC CellSaic(out)). The significant improvement of %NBGL of MSC CellSaic(in) compared to those of the MSC CellSaic(none) or MSC CellSaic(out) (*p* < 0.01) was confirmed. It is noteworthy that the number of islets required for treatment was reduced by the co-microencapsulation of the MSC CellSaics. From the stained sections, a larger number/area of blood vessels was observed around MSC CellSaic(in), which suggests that angiogenic factors secreted by the MSC CellSaics through the microcapsules can function locally for their enhanced efficacy.

## 2. Experimental Section

### 2.1. Animals

All animals were purchased from Charles River Japan (Yokohama, Japan). Rat islets were isolated from Wistar rats (7–8 weeks old, ♂). Balb/c (6–7 weeks old, ♂) and NOD/SCID (6–7 weeks old, ♂) mice were used as the recipients for islet transplantation. The experimental protocol was approved by the animal control committee of Fujifilm Corporation (A-1-190114, A-1-190113, approved on 5 April 2019)

Diabetic mouse models were prepared as follows: streptozotocin was injected into mouse intraperitoneal cavities (250 mg/kg body weight). One week after the injection, the nonfasting blood glucose level of each mouse was measured. The individual was recognized as diabetic when the glucose level was above 350 mg/dL for two consecutive days.

### 2.2. Materials

All reagents were purchased from Fujifilm Wako Pure Chemical (Osaka, Japan) unless otherwise stated. Medical-grade AL10 (Kimica, Tokyo, Japan) was used for alginate polymer. Alginate solution was prepared by dissolving AL10 in 10 mM MOPS buffer (Dojino, Kumamoto, Japan) to 3.9 *w*/*v*%, filtered through a 0.2 μm sterile filter (8-1020-050, Merck, Burlington, MA, USA). P2533 (Sigma-Aldrich, St. Louis, MO, USA) was used for poly-L-ornithine hydrochloride (PLO). PLO solution was prepared by dissolving P2533 in isotonic solution to 0.05 *w*/*v*%. After dissolution, filtration was performed using a Mustang E-membrane (0.2 µm, MSTG25E3, Pall, Port Washington, NY, USA).

### 2.3. Isolation of Rat Islets

Islets were isolated from rat pancreas using 1 mg/mL collagenase V (Sigma type V, Sigma Chemicals, St. Louis, MO, USA) solution in 10 mL HBSS through a common bile-duct catheter. After removal of spleen, duodenum, and stomach, the pancreas was digested (30 min, 37 °C). Thereafter, density gradient centrifugation was performed as previously described [29,30].

### 2.4. Preparation of MSC CellSaics

MSCs were generated as previously described [31]. Briefly, adipose tissue from a C57BL/6N was cut into small pieces and digested at 37 °C for 1 h with 2 mg/mL collagenase (Nitta Gelatin, Tokyo, Japan) in Dulbecco’s phosphate-buffered saline. The sample was centrifuged at 600× *g* for 5 min, washed, centrifuged again, and seeded into a flask containing MSCGM (PT-2501, Lonza, Basel, Switzerland). The medium used for MSCs was high-glucose DMEM containing 10% fetal bovine serum (30-2020, ATCC, Manassas, VA, USA) and penicillin streptomycin/amphotericin B. MSCs were cultured at 37 °C in a 5% CO_2_ humidified atmosphere. MSCs were negative for the endothelial markers 49e and CD117 and for the hematopoietic markers CD34, CD45, and CD11b. These cells expressed Sca-1, CD44, CD29, and CD90 [31]. RCP pieces were generated as previously described, and the petaloid pieces were used [13]. The MSC CellSaics were prepared by mixing MSCs (1.2 × 10^5^ cells/mL) and RCP pieces (0.25 mg/mL) in MSC medium; this mixture was seeded onto a 35 mm dish (EZ SPHERE 4000-903SP, AGC Techno Glass, Haibara, Japan).

### 2.5. Preparation of Alginate Microcapsules

Microcapsules were prepared using a commercially available encapsulator (Encapsulator B-390, Buchi Japan, Tokyo, Japan). An alginate solution was set on an encapsulator, nebulized into 100 mM calcium chloride or 20 mM barium chloride solution (wind speed: 2.5 NL/min, liquid flow rate: 1.0 mL/min), and stirred for 7 min (Ca^2+^ cross-linked, and Ba^2+^ cross-linked microcapsules). For PLO cross-linked microcapsules, Ca^2+^ cross-linked microcapsules were immersed in 0.05 *w*/*v*% PLO isotonic solution, shaken for 6 min on a rotator, and then washed twice with isotonic solution. This was followed by further immersing in 0.03 *w*/*v*% sodium alginate solution for 1 min and then washing again with isotonic solution.

Physical durability was evaluated by osmotic pressure test with reference to the literature [32], after which 10 mL of 0.45 *w*/*v*% NaCl solution was placed in a six well plate and approximately 100 microcapsules were added; after stirring with a rotator for 1 h, the number of broken capsules was counted under an optical microscope and the percentage was calculated.

The IgG-blocking performance was also evaluated with reference to the literature [25]. First, microcapsules containing magnetic particles were prepared by encapsulating 1.0 mL of alginate solution mixed with 1 μL of Dynabeads (goat anti-mouse IgG, Thermo Fisher Scientific, Waltham, MA, USA), as described above. Next, 3 μL of fluorescent antibody (Mouse IgG Isotype Control Alexa Fluor 488 Conjugated, bs-0296P-A488, Funakoshi, Tokyo, Japan) was diluted by adding 897 μL of isotonic solution. The fluorescent IgG diluent was shaded with aluminum foil. Following this, a microcapsule containing magnetic particles with 50 μL isotonic solution was added to a well of a 96 well glass bottom plate. Subsequently, 2.5 μL of fluorescent antibody diluent was added and the fluorescent spots inside the microcapsules were counted as a function of time under a fluorescence microscope (BZ-X710, Keyence, Osaka, Japan, magnification: 10×, GFP filter, reduced fading mode, exposure time 1 s).

### 2.6. Preparation of MSC CellSaic(in) and MSC CellSaic(None)

An alginate solution mixed with islets/MSC CellSaics was uniformly nebulized into 100 mM calcium chloride solution (wind speed: 2.5 NL/min, liquid flow rate: 1.0 mL/min) and stirred in 100 mM calcium chloride solution for 7 min, and the prepared microcapsules were collected and immersed in 0.05 *w*/*v*% PLO solution. They were shaken for 6 min on a rotator and then washed twice with isotonic solution. This were followed by further coating in 0.03 *w*/*v*% sodium alginate solution for 1 min and then washing again with isotonic solution. The prepared microcapsules were transferred to the transplantation medium (PNIM4, Cosmo Bio, Sapporo, Japan).

### 2.7. In Vitro Evaluations of MSC CellSaic(in) and MSC CellSaic(None)

Glucose-stimulated insulin secretion (GSIS) test was performed as follows. Cell culture inserts (Costar3421, Corning, Corning, NY, USA) were filled with islets or microencapsulated islets (approximately 30 pieces, 150 μL), and then, 400 μL of the low-glucose solution (Cosmo Bio Inc., Sapporo, Japan, PNIM4) was added to each well of a 24 well plate and each insert was soaked there for 1 h. The insert was then soaked again in a different well with new a 400 μL low-glucose solution, and after 1 h the insert was removed and the remaining liquid in the well was sampled (L). The removed insert was immediately transferred to a well containing 400 μL of 20 mM glucose solution (PNIMT, CosmoBio) and soaked for 1 h. The insert was then removed and the remaining solution in the well was sampled (H1). The concentrations of insulin in L and H1 solutions were quantified by ELISA (80-INSRT-E01, ALPCO, Salem, NH, USA).

The number of islets in the microcapsules was measured by visually counting all the islets from the Cell3imager (SCREEN, Kyoto, Japan) images.

Impurities were verified as follows: Limulus ES-II Single Test (Wako, Osaka, Japan, sensitivity: 0.03 EU/mL) was tested for endotoxin levels of solutions according to the manual. Mycoplasma denial tests were done using MycoAlert (Lonza, Basel, Switzerland). Sterility was checked as follows: 1 mL of the solution was seeded on SCD agar (TSA-3P, Sysmex-bioMérieux, Tokyo, Japan) and incubated at 27.5 °C for 1 week to check for the presence of colonies.

### 2.8. In Vivo Evaluations of MSC CellSaic(In), MSC CellSaic(None), and MSC CellSaic(Out)

Intraperitoneal transplantation was performed as follows. Under isoflurane anesthesia, the skin and muscles of the abdomen were incised by about 1 cm. Microcapsules or MSC CellSaics were then aspirated with a pipette and the entire dose was administered intraperitoneally. After administration, muscle and skin were sutured.

Subcutaneous transplantation was performed as follows. Under isoflurane anesthesia, the dorsal skin around the hindfoot was incised by about 1 cm, and the internal fibers were cut with scissors to the forefoot to make a space. The graft was aspirated with a pipette and the tip was inserted into the back of the incised space to administer the entire dose. The skin was sutured so that the administered fluid did not spill out.

For nonfasting blood glucose level (BGL) measurement, blood was collected from the tail vein. BGL was measured every weekday using LAB Gluco (Foracare Japan Co., Ltd., Tokyo, Japan). If the BGL was above 250 mg/dL for two consecutive days, it was considered to be a graft failure, and %NBGL was calculated. Body weights were also evaluated at the same time as BGL. After 28 days following transplantation, mice were sacrificed and the peripheries of transplants were removed and fixed in a 10% phosphate-buffered formalin solution. Hematoxlylin and Eosin (H&E)-stained sections were prepared for histological examination. The average number and the area of blood vessels around a microcapsule were measured using the “measure” command in imageJ software (1.52v, NIH, Bethesda, MD, USA). To assess the tissue response, immune cells around the microcapsules were observed and scored by a pathologist and a veterinarian at FUJIFILM Corp. For immunostaining, deparaffinized sections were first incubated with anti-insulin antibodies (I2018, Sigma-Aldrich) and then with LSAB2 biotin-conjugated secondary antibodies (K1015, Dako, Glostrup, Denmark) plus streptavidin–horseradish peroxidase (K1016, Dako). Next, signals were developed using diaminobenzidine solution (K3468, Dako), and sections were then counterstained with hematoxylin.

### 2.9. Statistical Analyses

The results are presented as means ± standard deviation. The average BGL for 28 days after the transplantation, and the average number and area of blood vessels around a microcapsule were analyzed using two-way analysis of variance with Welch’s t-test (Microsoft Excel 2007). %NBGL was analyzed with log-rank test (Prism 5, GraphPad Software, San Diego, CA, USA). *p* < 0.05 was considered to be statistically significant.

## 3. Results

### 3.1. Preparation of Immuno-Isolating Microcapsules

#### 3.1.1. Selection of Cross-Linking Agents for Alginate Microcapsules

In order to select the optimal cross-linking agent for microcapsules with feasible immuno-isolating ability, in vitro evaluations were performed for alginate microcapsules prepared with three different cross-linking agents, namely Ca^2+^, Ba^2+^, and PLO [22,23,24]. The conditions used to produce the microcapsules were in accordance with those typical in the literatures.

First, resistances against mechanical force were compared through the osmotic pressure test [32]. As a result, the complete melting of the microcapsules cross-linked with Ca^2+^ was observed, while those cross-linked with Ba^2+^ and PLO were not damaged at all (Table 1). Second, IgG blocking abilities were evaluated [25]. It was shown that IgGs readily permeated the microcapsules after only 30 min for Ba^2+^, whereas no permeation was detected for PLO even after 120 min (Table 1, Figure S1a,b). Therefore, PLO was selected as the cross-linker to formulate alginate microcapsules for use in subsequent experiments.

**Figure 1:**
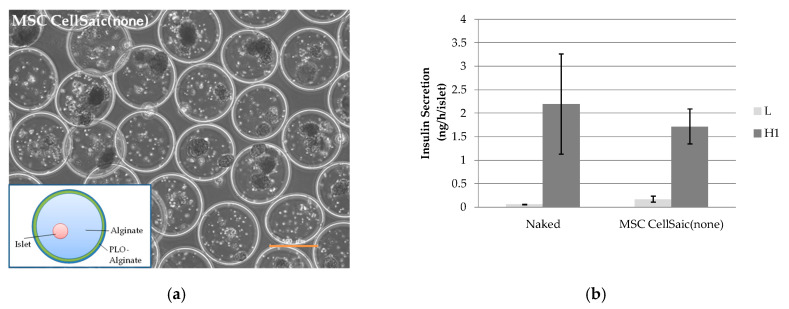
In vitro evaluations of MSC CellSaic(none). (**a**) An optical microscopy image of MSC CellSaic(none). The scale bar indicates 500 μm. (**b**) Insulin secretion from one islet per hour for naked islets and MSC CellSaic(none) microcapsules, evaluated by GSIS test with the insert-cell method (mean ± standard deviation, *n* = 3). L: insulin secretion in 3 mM glucose solution, H1: insulin secretion in 20 mM glucose solution.

PLO cross-linked alginate microcapsules encapsulating rat islets (MSC CellSaic(none)) were prepared (Figure 1a) and insulin permeation through the microcapsule was evaluated via a glucose-stimulated insulin secretion (GSIS) test. The insulin secretion 1 h after stimulation with high-concentration glucose (20 mM) was 1.7 ± 0.4 ng/h/islet for MSC CellSaic(none), whereas that for naked islets was 2.2 ± 1.1 ng/h/islet (Figure 1b). Although insulin secretion turned out to be slightly lower for MSC CellSaic(none) than naked islets, it affirmed the secretion of insulin permeating through the microcapsules. Note that the stimulation index (SI) value, which indicates the insulin secretion ratio by 20 mM glucose stimulation to 3 mM glucose (nonstimulation), of MSC CellSaic(none) was 10 ± 1.4, which is comparable to the value reported elsewhere [33].

Endotoxin, sterility, and mycoplasma denial tests were performed on alginate solution, PLO solution, washing solution, cross-linking solution, and islet microcapsule supernatants, respectively, and none were detected.

#### 3.1.2. In Vivo Evaluation of MSC CellSaic (None)

MSC CellSaic(none) was transplanted intraperitoneally or subcutaneously into diabetic balb/c model mice, and the average non-fasting blood glucose level (BGL) and percentage of the normal glucose level rate (%NBGL) were monitored for the following 28 days.

**Figure 2:**
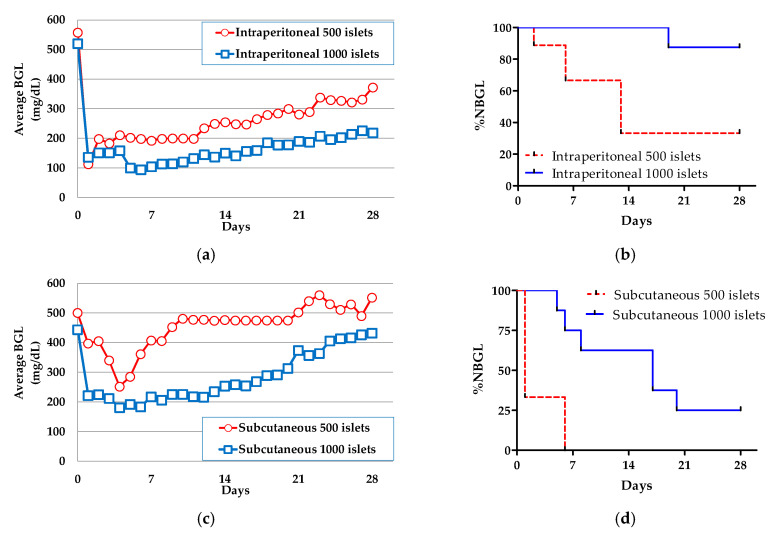
Intraperitoneal and subcutaneous transplantation of MSC CellSaic(none) into diabetic balb/c mice and subsequent monitoring. (**a**) Average nonfasting blood glucose level (average BGL) of intraperitoneal transplantation (1000 islets: □: *n* = 8, 500 islets: ○: *n* = 9). (**b**) Percentage of the normal glucose level rate (%NBGL) following intraperitoneal transplantation (1000 islets: solid line, 500 islets: dashed line). (**c**) Average BGL following subcutaneous transplantation (1000 islets: □: *n* = 9, 500 islets: ○: *n* = 3). (**d**) %NBGL following subcutaneous transplantation (1000 islets: solid line, 500 islets: dashed line).

The results for intraperitoneal transplantation are shown in Figure 2a,b. When 1000 islets were transplanted, the average BGL and %NBGL values at Day 28 were 219 ± 106 mg/dL and 87.5% (seven of eight samples), respectively. When the number of islets was decreased to 500, the average BGL increased to 372 ± 177 mg/dL and %NBGL decreased to 33% (three of nine samples).

The results for subcutaneous transplantation are shown in Figure 2c,d. In the case of transplantation of 1000 islets, the average BGL was 432 ± 138 mg/dL and the %NBGL was 25% (two of nine samples) at Day 28. In the same manner as the intraperitoneal transplantation, the values changed for transplantation of 500 islets to 552 ± 40.6 mg/dL and 0% (zero of three samples), respectively. Apparently, the %NBGL of subcutaneous transplantation was inferior to that of intraperitoneal. This was presumed to be because of the low density of blood vessels at the subcutaneous site and strong inflammatory responses [11]. Thus, we proceeded to prepare alginate microcapsules which co-encapsulated the MSC CellSaics and rat islets (MSC CellSain(in)) as a strategy to increase the number of blood vessels and reduce inflammatory responses in the periphery for applications in subcutaneous transplantation.

### 3.2. The Effect of Co-Microencapusulation of the MSC CellSaics and Islets Inside on %NBGL

#### 3.2.1. Preparation of MSC CellSaic(in)

**Figure 3:**
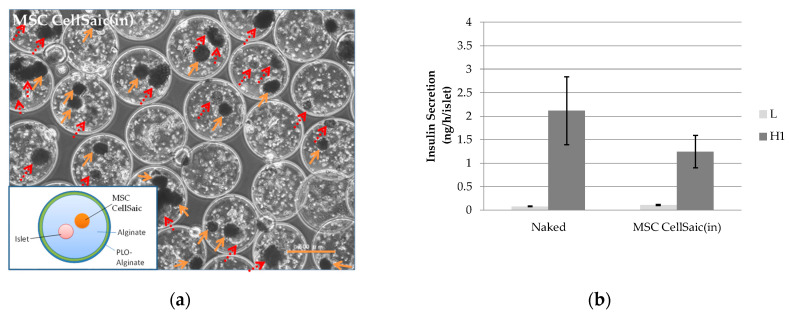
In vitro evaluation of MSC CellSaic(in). (**a**) An optical micrograph image. Scale bar indicates 500 μm. Solid arrows indicate MSC CellSaics, and dotted arrows indicate rat islets. (**b**) Insulin secretion from one islet per hour for naked islets and MSC CellSaic(in) microcapsules, evaluated by GSIS test with the insert-cell method (mean ± standard deviation, *n* = 3). L: insulin secretion in 3 mM glucose solution, H1: insulin secretion in 20 mM glucose solution.

To confirm the co-microencapsulation effect of the MSC CellSaics with islets, MSC CellSaic(in) was prepared (Figure 3a). Note that endotoxin, sterility, and mycoplasma denial tests were performed for the supernatant solution of the prepared microcapsules, and none were detected.

As the GSIS test was performed, the insulin secretion rate of MSC CellSaic(in) at 1 h after stimulation with a high concentration of glucose (20 mM) was determined to be 1.2 ± 0.3 ng/h/islet (Figure 3b). Although this was, again, slightly lower than the secretion by the naked islets on the same day (2.1 ± 0.7 ng/h/islet), the secretion capability was affirmed with an SI value of 11 ± 2.3.

#### 3.2.2. In Vivo Evaluations of MSC CellSaic(in) and MSC CellSaic(out)

**Figure 4:**
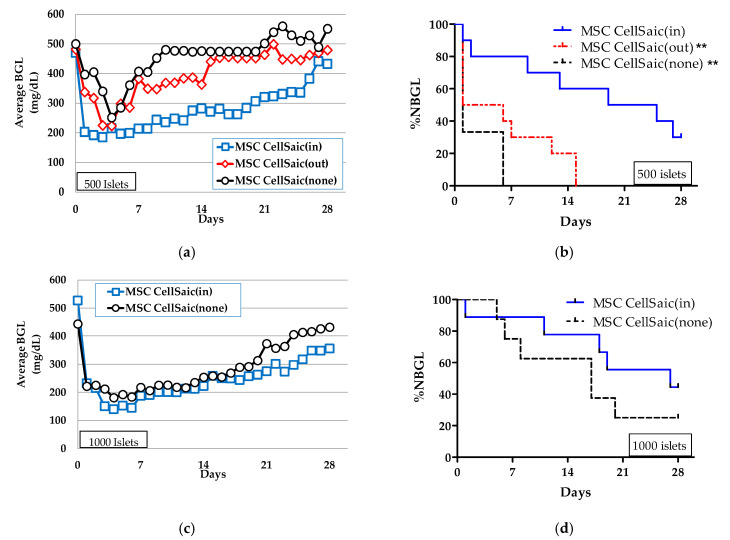
Subcutaneous transplantation of MSC CellSaic(none), MSC CellSaic(out), and MSC CellSaic(in) microcapsules into diabetic balb/c mice and subsequent monitoring. MSC CellSaic(out) was prepared by adding equal numbers of MSC CellSaics to islets outside of MSC CellSaic(none). (**a**) Average BGL following 500 islet transplantation (MSC CellSaic(none) ○: *n* = 9, MSC CellSaic(out): ◇: *n* = 10, MSC CellSaic(in): □: *n* = 10). (**b**) %NBGL following 500 islet transplantation (MSC CellSaic(none): dashed line, MSC CellSaic(out): dotted line, MSC CellSaic(in): solid line). (**c**) Average BGL following 1000 islet transplantation. (MSC CellSaic(none): ○: *n* = 8, MSC CellSaic(in): □: *n* = 10.) ** means that %NBGL of MSC CellSaic(in) was higher than those of MSC CellSaic(none) or MSC CellSaic(out) with *p* < 0.01. (**d**) %NBGL following 1000 islet transplantation (MSC CellSaic(none): dashed line, MSC CellSaic(in): solid line).

Average BGL and %NBGL values after subcutaneous transplantation of MSC CellSaic(in) were compared to those of MSC CellSaic(none) (Figure 4). As 500 islets were transplanted (Figure 4a,b), the average BGL of MSC CellSaic(in) 28 days after transplantation was lower than that of MSC CellSaic(none) with *p* < 0.05, and %NBGL of MSC CellSaic(in) was higher than that of MSC CellSaic(none) with *p* < 0.01.

As 1000 islets were transplanted (Figure 4c,d), MSC CellSaic(in) also showed an improvement compared to MSC CellSaic(none) in the same manner. It is noteworthy, the performance of MSC CellSaic(in) with 500 islets surpassed that of MSC CellSaic(none) with 1000 islets. Intraperitoneal glucose tolerance test (IPGTT) was conducted on euglycemic 3 mice 28 days post-subcutaneous transplant of MSC CellSaic(in) (Figure S2). Glucose clearance of them was similar to that of a non-diabetic control at all time points after glucose loading. Thus, we thought that at 28 days after subcuntaneousl transplantation islets in MSC CellSaic(in) could secrete insulin response to glucoseFurthermore, to differentially evaluate the co-existing effect and co-encapsulating effect of the MSC CellSaics, the MSC CellSaics were vicinally transplanted with MSC CellSaic(none) (MSC CellSaic(out)). Regarding the results of the average BGL and the %NBGL evaluations for MSC CellSaic(out), although they showed better performance compared to MSC CellSaic(none), they did not reach the performance of MSC CellSaic(in) (Figure 4a,b). The superior performance of MSC CellSaic(in) among the three supported our approach of co-encapsulating the MSC CellSaics with islets for subcutaneous applications.

#### 3.2.3. Comparison of Post-Implantation Section Images

**Figure 5:**
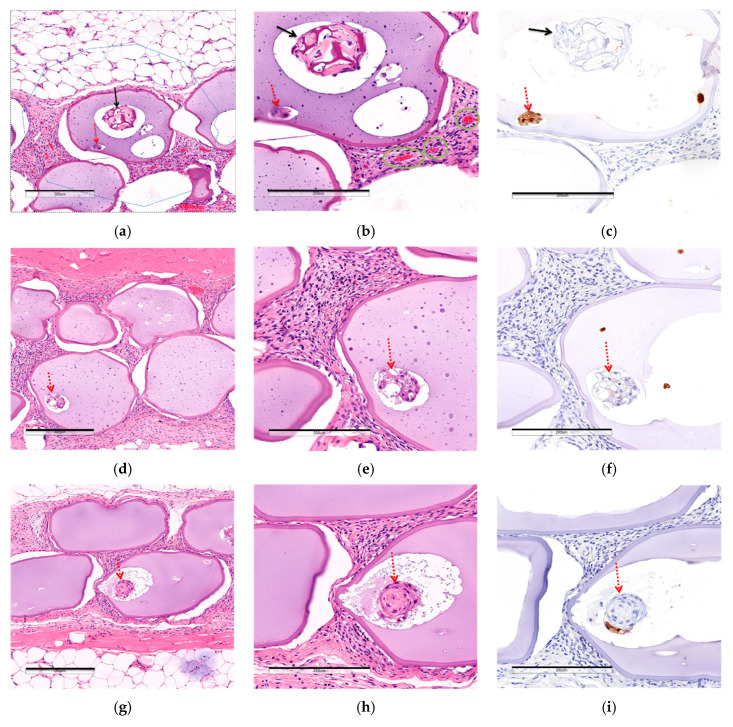

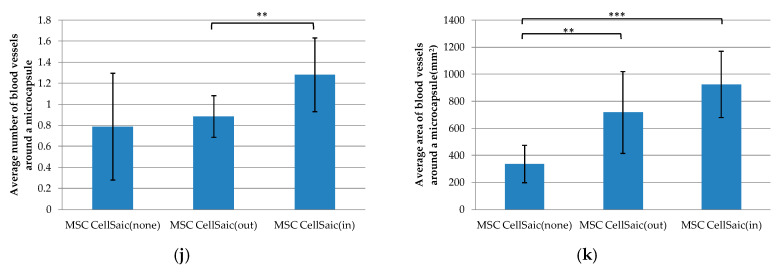
H&E staining or insulin immuno-staining sections of microcapsules 28 days after subcutaneous transplantation. Solid arrows indicate MSC CellSaic. Dotted arrows indicate an islet. (**a**–**c**) MSC CellSaic(in), (**d**–**f**) MSC CellSaic(out), and (**g**–**i**) MSC CellSaic(none). (**a**,**d**,**g**) H&E-staining images (×7). Scale bars indicate 300 µm. (**a**) A blue dotted line represents the 200 μm surrounding the microcapsules. (**b**,**e**,**h**) H&E-staining images (×10). Scale bars indicate 200 µm. The thick green solid line represents the blood vessels around a microcapsule. (**c**,**f**,**i**) Insulin immuno-staining images (×10). Scale bars indicate 200 µm. (**j**) Interspecimen mean ± standard deviation of the mean number of vessels in the 200 μm around the microcapsules. (**k**) Interspecimen mean ± standard deviation of the mean vascular area 200 μm around one microcapsule. **, *** mean that *p* < 0.01, *p* < 0.005, respectively with 2 way analysis of Welch’s t-test.

To investigate the hypothesis that the location of the MSC CellSaics affected their efficacy, the number and the area of blood vessels within 200 μm of the boundaries of microcapsules after 28 days of transplantation were comapared by examining H&E-stained sections (Figure 5). As a result, MSC CellSaic(in) had 1.28 ± 0.35 blood vessels and a 920 ± 250 μm^2^ blood vessel area around each microcapsule, whereas MSC CellSaic(out) had 0.88 ± 0.20 and 717 ± 302 μm^2^, and MSC CellSaic(none) had 0.79 ± 0.51 and 336 ± 139 µm^2^, respectively. This suggested a larger number and area of blood vessels around MSC CellSaic(in), which might be a key factor in its supreme efficiency.

Additionally, inflammatory and foreign-body responses around the microcapsules were analyzed by examination by a pathologist of the same H&E-stained sections as above, but some immune cells (neutrophils, macrophages, lymphosites) and fibroblasts were observed around MSC CellSaic(in), MSC CellSaic(out), and MSC CellSaic(none), respectively, and no significant differences were found. We suspected that the immune response could not be completely avoided by microencapsulation and MSC CellSaics. In order to verify this, MSCCellSaic(in) was transplanted into subcutaneous diabetic NOD/SCID mice and %NBGL was analyzed (Figure S3). They reached 100% %NBGL after 28 days. These results indicated that the combination of microencapsulation and MSC CellSaic was not enough for immunoisolation of transplanted islets.

Although not stated above, the effects of the MSC CellSaics following intraperitoneal as well as subcutaneous transplantation were evaluated (Figure S4). Similarly to subcutaneous transplantation, MSC CellSaic(in) and MSC CellSaic(out) both demonstrated improved efficiency compared to MSC CellSaic(none). However, unlike subcutaneous transplantation, the difference between MSC CellSaic(in) and MSC CellSaic(out) was not measurable following intraperitoneal transplantation.

## 4. Discussion

**Figure 6:**
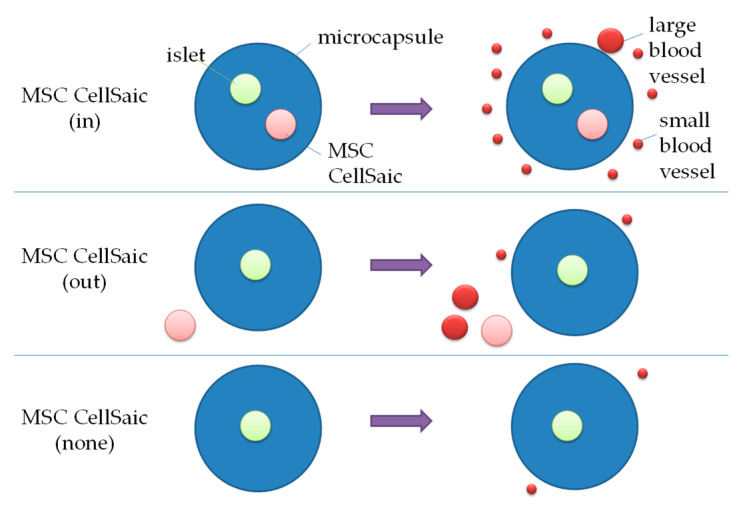
The trend of angiogenesis depending on the position of MSC CellSaics.

The trend of angiogenesis around microcapsules, depending on the position of MSC CellSaics, is shown in Figure 6. Related to this point, we previously reported that the MSC CellSaics secrete higher levels of angiogenic factors, including angiogenesin (14 kDa), bFGF (18 kDa), IGF-1 (8 kDa), and VEGF (20 kDa), than MSCs [14]. These angiogenic factors are larger than insulin (6 kDa) but would be smaller than an IgG antibody (160 kDa), and small enough to permeate the microcapsules. Based on this, MSC CellSaic(in) should be capable of locally inducing blood vessels around the capsule, thus enabling the reversal of BGL with a lower number of islets. In contrast, for MSC CellSaic(out), the MSC CellSaics were presumed to be located too far from the microcapsules to induce the blood vessels around them, which could be considered a main cause of insufficient blood vessels for BGL normalization.

A method of forming a vascular bed subcutaneously before transplantation and good blood glucose control by transplanting islets there has been reported [10]. In this study, we could induce blood vessels around microcapsules by MSC CellSaic(in) without making a vascular bed and control blood glucose level, although %NBGL was not 100%. We think that use of co-microencapsulating of MSC CellSaics could give a simpler transplantation method.

On the other hand, an anti-inflammatory effect was also initially expected as an MSC CellSaic effect, but this was not confirmed in this study. It is known that MSCs secrete anti-inflammatory cytokines (IL-10, TGF-α, IDO, TSG-6, etc.) [15,16,17,31], and we have confirmed that MSC CellSaics secreted 3.1-fold more anti-inflammatory cytokine TSG-6 in response to concentration of inflammatory cytokine TNF-α than the MSC spheroids [20]. In reference [34], it is reported that transplantation of ALG-PLO microencapsulated islets trigger inflammation by increasing the TNF-α concentration at the transplantation site. So we hoped that MSC CellSaics could suppress the above inflammation by TSG-6 released from MSC CellSaics. In addition, MSCs were alive after 28 days transplantation from H&E staining of the tissue section since MSC CellSaics maintained its shape and also the nucleuses were stained by hematoxylin. Therefore, we thought that the amount of anti-inflammatory cytokines secreted from MSC CellSaics was not sufficient for suppression of the immune response to ALG-PLO microcapsules [26,34]. As future work, we would like to study about a method to increase the amount of anti-inflammatory cytokines from MSC CellSaics [35], or use of microcapsules with high biocompatibility [11,26,27,28] in order to improve %NBGL.

## 5. Conclusions

Microcapsules co-encapsulating MSC CellSaics and islets were prepared, and their therapeutic effect for diabetes following subcutaneous transplantation was evaluated in diabetic immunocompetent mice. Their normalization ability of blood glucose levels was superior to those of the microcapsules without the MSC CellSaics, or the MSC CellSaics transplanted outside the microcapsules. Local angiogenesis was characteristically observed around the microcapsules containing MSC CellSaics and islets, which suggests that angiogenic factors secreted by the MSC CellSaics through the microcapsules can function locally for their enhanced efficacy.

## Figures and Tables

**Table 1:** In vitro evaluations of microcapsules with different cross-linking agents.

Microcapsule Constitution	Resistance against Osmotic Pressure (%)	Duration Time for IgG Blocking (min)
Alginate/Ca^2+^	0	10
Alginate/Ba^2+^	100	30
Alginate/PLO	100	≥120

## Supplementary Materials

Supplementary Materials can be found at https://www.mdpi.com/2227-9059/8/9/318/s1, Figure S1: IgG blocking performance of alginate microcapsules with different cross-linking agents, Figure S2: IGPTT of MSC CellSaic(in) (euglycemia), Figure S3: Blood glucose levels in subcutaneous transplantation of CellSaic(in) in a diabetic NOD/SCID mouse models, Figure S4: Intraperitoneal transplantations of MSC CellSaic(none), MSC CellSaic(out), and MSC CellSaic(in).
