# Supplementary material for: Co-Microencapsulation of Islets and MSC CellSaics, Mosaic-Like Aggregates of MSCs and Recombinant Peptide Pieces, and Therapeutic Effects of Their Subcutaneous Transplantation on Diabetes
Source: Biomedicines. 2020 Aug 31;8(9):318. doi: 10.3390/biomedicines8090318 (PMC7554936; doi:10.3390/biomedicines8090318)
Supplement: Supplementary file 1 [file biomedicines-08-00318-s001.zip › biomedicines-883942-supplementary.pdf]

biomedicines

MDPI

Co-Microencapsulation of Islets and MSC CellSaics, Mosaic-Like Aggregates of MSCs and Recombinant Peptide Pieces, and Therapeutic Effects of Their Subcutaneous Transplantation on Diabetes

Yusuke Mochizuki, Ryo Kogawa, Ryuta Takegami, Kentaro Nakamura, Akira Wakabayashi, Tadashi Ito and Yasuhiro Yoshioka

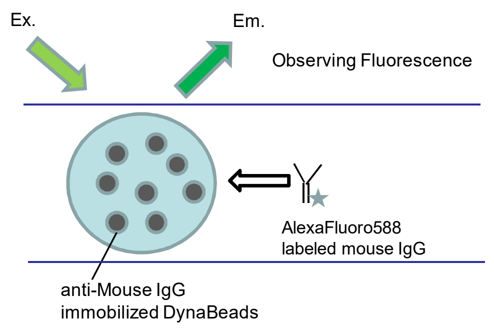

(a)

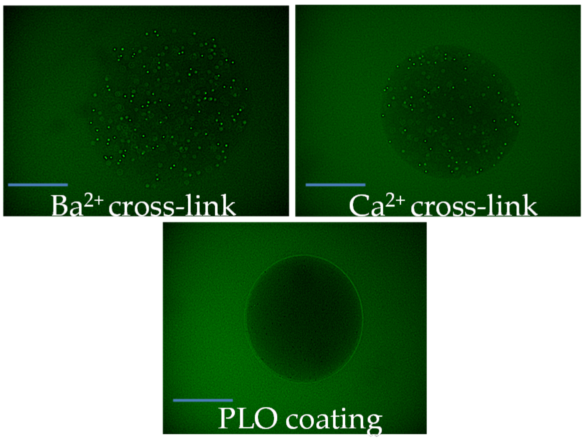

(b)

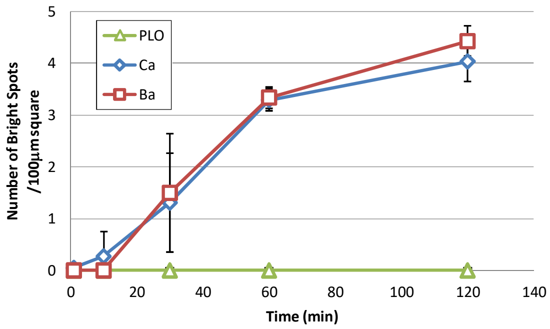

(c)

Figure S1. IgG blocking performance of alginate microcapsules with different cross-linking agents. (**a**) The method for measuring the IgG blocking performance of microcapsules. anti-mouse IgG-immobilized DynaBeads are encapsulated in microcapsules, and when AlexaFluoro588-modified mouse IgG enters from outside, it accumulates on DynaBeads and is observed fluorescently. (**b**) Fluorescence microscopy images after 120 min of injection of fluorescence-modified IgG (top left of photo) Ba^{2+} cross-linked microcapsules (top right) Ca^{2+} cross-linked microcapsules and (bottom) poly-L-ornithine cross-linked capsules. Scale bars indicate 300 µm. (**c**) The time course of the number of fluorescing magnetic particles per unit area. Each was conducted with $n = 3$, and each plot represents a mean value ± standard deviation.

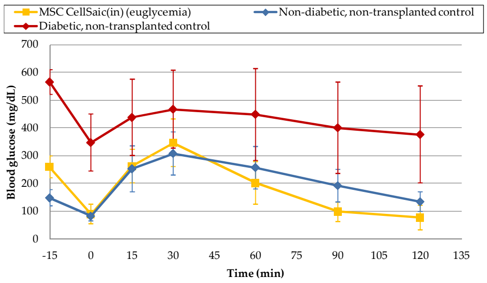

(a)

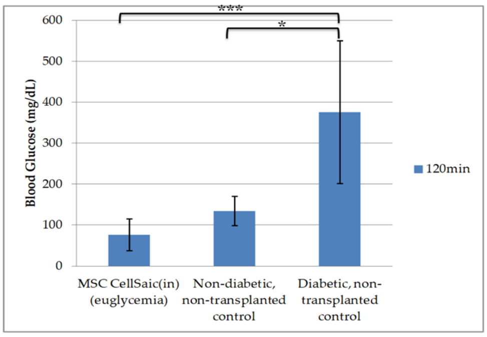

(b)

Figure S2. IGPTT of MSC CellSaic(in) (euglycemia). IPGTT were conducted on euglycemic 3 mice 28 days after subcutaneous transplantation of MSC CellSaic(in). Mice were fasted overnight prior to receiving an intraperitoneal glucose solution (2g/kg). (**a**) Blood glucose levels were evaluated before injection (-15 min), at baseline (time 0 min), 15, 30, 60, 90 and 120 min post-injection. Non-deabetic, non-transplanted control (n = 20), Diabetic, non-transplanted control (n = 7), (**b**) 120 minutes after glucose injection, blood glucose level from 'Cell Saic(in)(euglymemia)' and 'non-diabetic, non-transplanted control' had a significant difference in that from 'diabetic, non-transplanted control'.

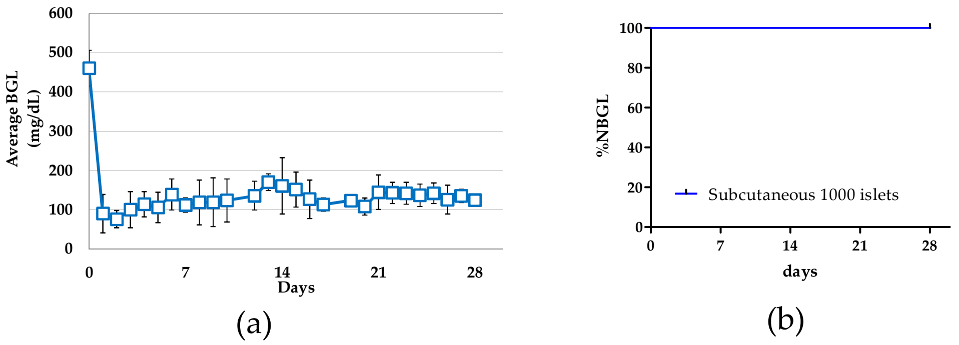

Figure S3. Blood glucose levels in subcutaneous transplantation of CellSaic(in) in a diabetic NOD/SCID mouse models CellSaic(in) containing 1000 islets were transplanted (*n* = 4). (**a**) Average BGL. (**b**) %NGBL.

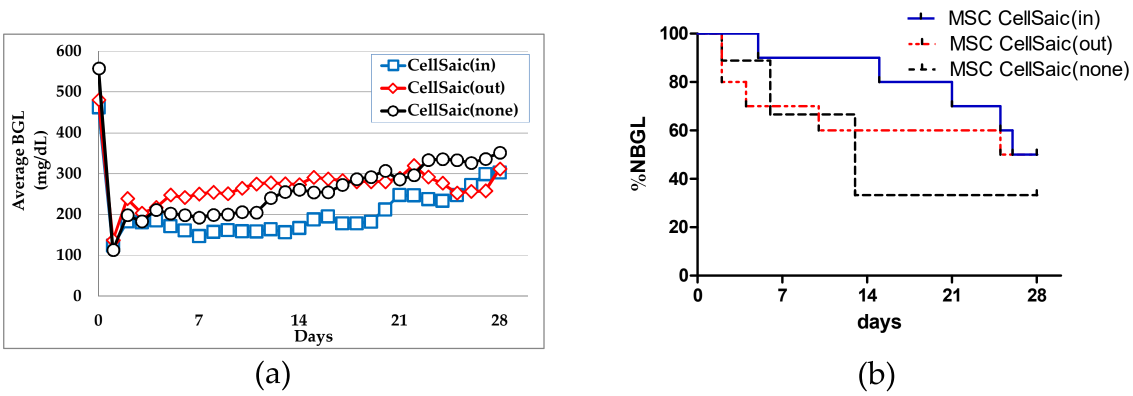

Figure S4. Intraperitoneal transplantations of MSC CellSaic(-), MSC CellSaic(out), and MSC CellSaic(in) microcapsules into diabetic balb/c mice and following monitoring. (**a**) Average BGL of 500 islets transplantation(MSC CellSaic(-)○: *n* = 9, MSC CellSaic(out): ◇:*n* = 10, MSC CellSaic(in): □:*n* = 10). (**b**) %NBGL of 500 islets transplantation (MSC CellSaic(-): dashed line, MSC CellSaic(out): dotted line, MSC CellSaic(in): solid line).
